# Isolation and genetic characterization of a mammalian orthoreovirus from *Vespertilio sinensis* in Japan

**DOI:** 10.1007/s00705-023-05782-x

**Published:** 2023-05-20

**Authors:** Ayano Ichikawa, Misa Katayama, Hayden Lai, Sekine Wataru, Akiko Takenaka-Uema, Taisuke Horimoto, Shin Murakami

**Affiliations:** Laboratory of Veterinary Microbiology, Graduate School of Agricultural and Life Sciences, The University of Tokyo, 1-1-1 Yayoi, Bunkyo-ku, Tokyo, 113-8657 Japan

## Abstract

Throughout East Asia, Europe, and North America, mammalian orthoreovirus (MRV), for which bats have been proposed to be natural reservoirs, has been detected in a variety of domestic and wild mammals, as well as in humans. Here, we isolated a novel MRV strain (designated as Kj22-33) from a fecal sample from *Vespertilio sinensis* bats in Japan. Strain Kj22-33 has a 10-segmented genome with a total length of 23,580 base pairs. Phylogenetic analysis indicated that Kj22-33 is a serotype 2 strain, the segmented genome of which has undergone reassortment with that of other MRV strains.

Reoviruses, members of the order *Reovirales*, are classified into two families, namely, *Sedoreoviridae* and *Spinareoviridae*, each of which comprises several genera. Organisms within a diverse spectrum of taxonomic groups, including protists, plants, fish, amphibians, birds, and mammals, serve as hosts for viruses from both of these families. *Mammalian orthoreovirus* is one of the 10 species comprising the genus *Orthoreovirus* within the family *Spinareoviridae*, and it can be further divided into four major serotypes (MRV1–4) based on antigenicity in neutralization and hemagglutination inhibition tests [1, 2].

Mammalian orthoreovirus (MRV) is a non-enveloped virus with a 10-segmented double-stranded RNA genome consisting of three large (L1–L3), three medium (M1–M3), and four small (S1–S4) segments. The L1, L2, L3, M1, M2, S1, S2, and S4 segments encode eight structural proteins (λ1, λ2, λ3, µ1, µ2, σ1, σ2, and σ3, respectively), whereas the M3 segment encodes two non-structural proteins (µNS and µNSC), and the S1 and S3 segments each encode a non-structural protein (σ1s and σNS, respectively). The external capsid protein σ1 plays a role in cell attachment and is the only determinant distinguishing the serotypes.

Since its initial isolation from the stools of children in 1954 [1], MRV has been reported in children with gastroenteritis [3–5]. In addition, strains of MRV have been detected continually in a wide range of mammals, including pigs [6], wild boars [7], cats [8], dogs [9], bats [10–17], and deer [18], and have also been found in wastewater [19], indicating the broad host range of this virus. Bats are particularly noteworthy in this regard, in that they are considered to be natural reservoirs of MRV, and a genetically diverse range of strains have been detected in different bat species [10]. However, although bat MRVs have been reported in multiple regions, including Europe [9, 11], the United States [13], China [10, 14–16], and Korea [17], to date, none of these viruses have been detected in Japanese bats, despite the presence of MRV in several mammals in Japan [6–9]. In this study, we detected and isolated a novel MRV strain from bats in Japan.

To determine whether bat MRVs are present in Japan, in July 2022, we collected fecal samples from Asian particolored bats (*Vespertilio sinensis*) in Saitama Prefecture, Japan. Suspensions of the collected feces were subsequently used to inoculate Vero cells expressing transmembrane serine protease 2 (Vero/TMPRSS2), which may support the replication of unknown viruses, including MRVs [20]. The inoculated cells were incubated at 37℃ in a 5% CO_2_ atmosphere, and at 3 days post-inoculation, we observed a clear cytopathic effect (CPE). The cell supernatant was passed through a 0.22-µm-pore-size filter, and the filtrate was used inoculate fresh Vero/TMPRSS2 cells. Consistently, we observed a CPE, thereby confirming the isolation of a virus (hereafter referred to as Kj22-33). To identify the viral genome type, we used two antiviral drugs – ribavirin and 5-iodo-2ʹ-deoxyuridine (IUDR) – which inhibit the growth of RNA and DNA viruses, respectively. Viral growth was found to be inhibited only in the presence of ribavirin, indicating that Kj22-33 has an RNA genome.

We purified the Kj22-33 isolate from the infected cell supernatant by ultracentrifugation with a 20% sucrose cushion and extracted the RNA using ISOGEN-LS (Nippon Gene, Toyama, Japan). An MGIEasy RNA Directional Library Prep Set (MGI, Shenzhen, China) was used to prepare a library for genome sequencing using a DNBSEQ G400RS high-throughput sequencer (MGI, Shenzhen, China). The dataset thus obtained was assembled *de novo* using CLC Genomics Workbench software (ver. 8; CLCbio, Aarhus, Denmark), and accordingly, we obtained sequences for 10 segments. The 5ʹ and 3ʹ regions of each segment were subsequently determined by RACE (rapid amplification of cDNA ends) using a SMARTer RACE 5'/3' Kit (Takara Bio, Shiga, Japan), on the basis of which we determined the full viral genome sequence (GenBank accession numbers LC752173–LC752182). NCBI BLAST searches revealed that all 10 segments showed high similarity (96.2% to 99.5% identity) to the genomic sequences of MRV strains isolated from different animal species (Table 1).

**Table 1:** Mammalian orthoreovirus strains with the highest sequence similarity to each genome segment of Kj22-33 by NCBI BLAST search

Segment	% Identity	Serotype	Strain	Host	Country	Year	Accession no.
L1	98.3	T2	17-EF40	Bat	USA	2017	MW718862
L2	98.5	T2	WIV4	Bat	China	2011	KT444533
L3	98.9	T2	OV204	Deer	USA	2016	MK092966
M1	98.0	T1	B19-02	Bat	South Korea	2019	MW582625
M2	99.2	T1	B19-02	Bat	South Korea	2019	MW582626
M3	99.0	T2	WIV3	Bat	China	2011	KT444577
S1	96.2	T2	RpMRV-YN2012	Bat	China	2012	KM087111
S2	98.5	T2	OV204	Deer	USA	2016	MK092971
S3	99.1	T3	SD-14	Mink	China	2014	KT224512
S4	99.5	T2	WIV5	Bat	China	2011	KT444551

Phylogenetic analysis was performed based on sequences of each Kj22-33 genome segment (Fig. 1). Trees were constructed using the maximum-likelihood method based on the Tamura-Nei model in MEGA X [21]. The S1 sequence of Kj22-33 was found to group in the same clade as serotype 2 strains. However, although the S1 segment of Kj22-33 appears to be closely related to that of the strain MRV2/RpMRV-YN2012/bat/2012/China (YN2012), the other segments of Kj22-33 were observed to cluster in clades distant from that containing YN2012. MRV2/OV204/deer/2016/USA showed the highest nucleotide sequence similarity in the L3 and S2 genomic segments, but it also exhibited phylogenetic relatedness in the S2, S3, M3, L1, and L3 segments, suggesting an evolutionary association among these genomic segments. These findings suggest that Kj22-33 may have arisen as a consequence of genomic reassortment with the closely related YN2012 and OV204 strains, as well as others.

**Fig. 1:**
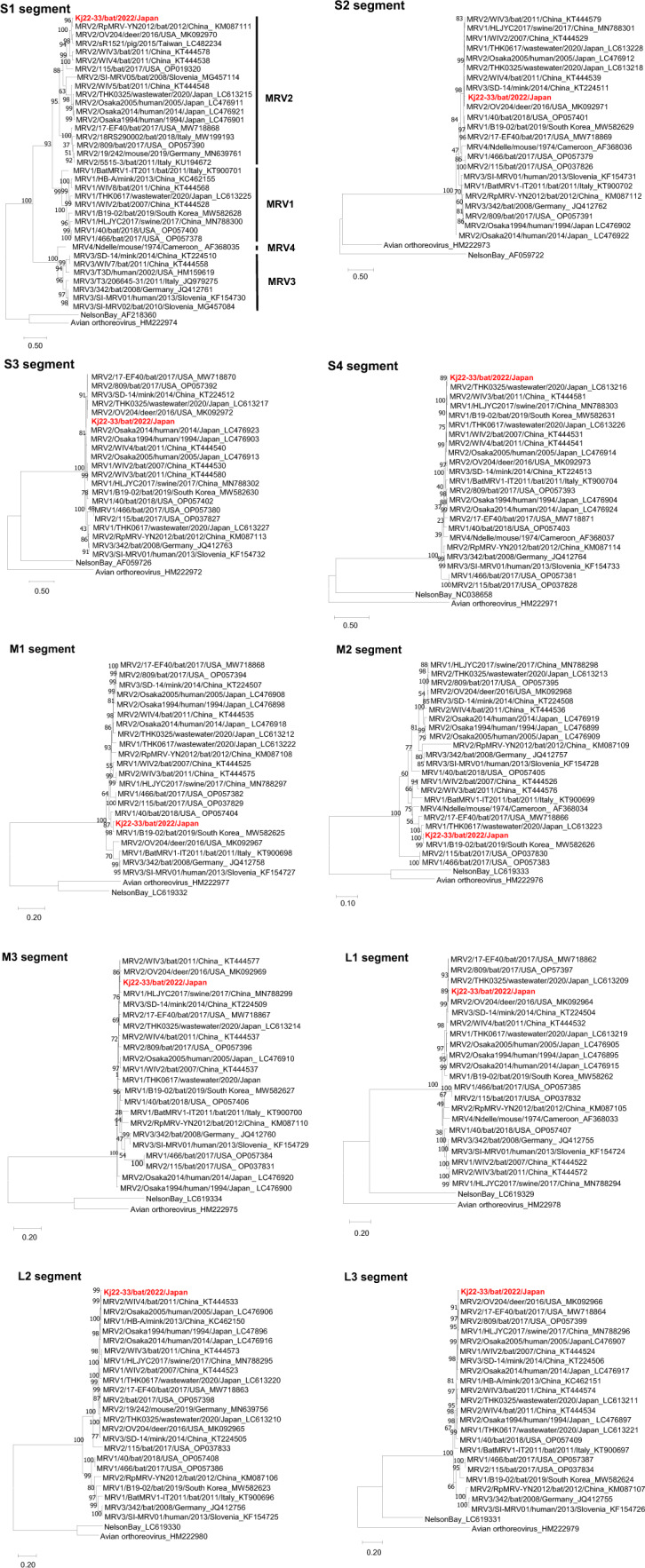
Phylogenetic analysis of the 10 genome segments of strain Kj22-33. Phylogenetic trees were constructed by the maximum-likelihood method with 1,000 bootstrap replicates using MEGA X software. The numbers at nodes denote bootstrap values based on 1000 replicates. The scale bar shows the evolutionary distance in terms of nucleotide substitutions per site. The virus strain Kj22-33 is shown in bold red. Virus strains are labeled as follows: MRV serotype/strain name/detection host or material/detection year/country/GenBank accession number. The four serotype groups are indicated on the right-hand side of the S1 segment tree.

In general, viruses exhibit a high degree of host specificity and are able to infect only a narrow range of hosts. In contrast, MRV has been established to have low host specificity and is accordingly characterized by a broad host range. In this study, we conducted a comprehensive phylogenetic analysis of all genomic segments and found that although segment S1 of the Kj22-33 isolate is closely related to the corresponding segment of the YN2012 strain obtained from Chinese bats, five out of the ten segments of Kj22-33 were most closely related to those of MRV identified in white-tailed deer in the USA, despite the large geographical distance. Given that the host of Kj22-33, the bat *Vespertilio sinensis*, is a common species in East Asia, and that it inhabits an environment quite distinct from that of the white-tailed deer, the reason for the apparently close relationship between these two geographically distant viruses remains unclear. Moreover, given its low host specificity, the global distribution and epidemiology of MRV remains largely undetermined, and further studies are therefore required to clarify its mode of spread.

## Data Availability

The complete genome sequences of the MRV strain identified in this study have been deposited in the GenBank database under the accession numbers LC752173–LC752182.
